# Supplementary material for: Causal Relationship between Aging and Anorexia Nervosa: A White-Matter-Microstructure-Mediated Mendelian Randomization Analysis
Source: Biomedicines. 2024 Aug 16;12(8):1874. doi: 10.3390/biomedicines12081874 (PMC11351342; doi:10.3390/biomedicines12081874)
Supplement: Supplementary file 1 [file biomedicines-12-01874-s001.zip › Supplementary Figure/Figure S4/Singe SNP plot of MR analysis for TL to ubm-b-1886.pdf]

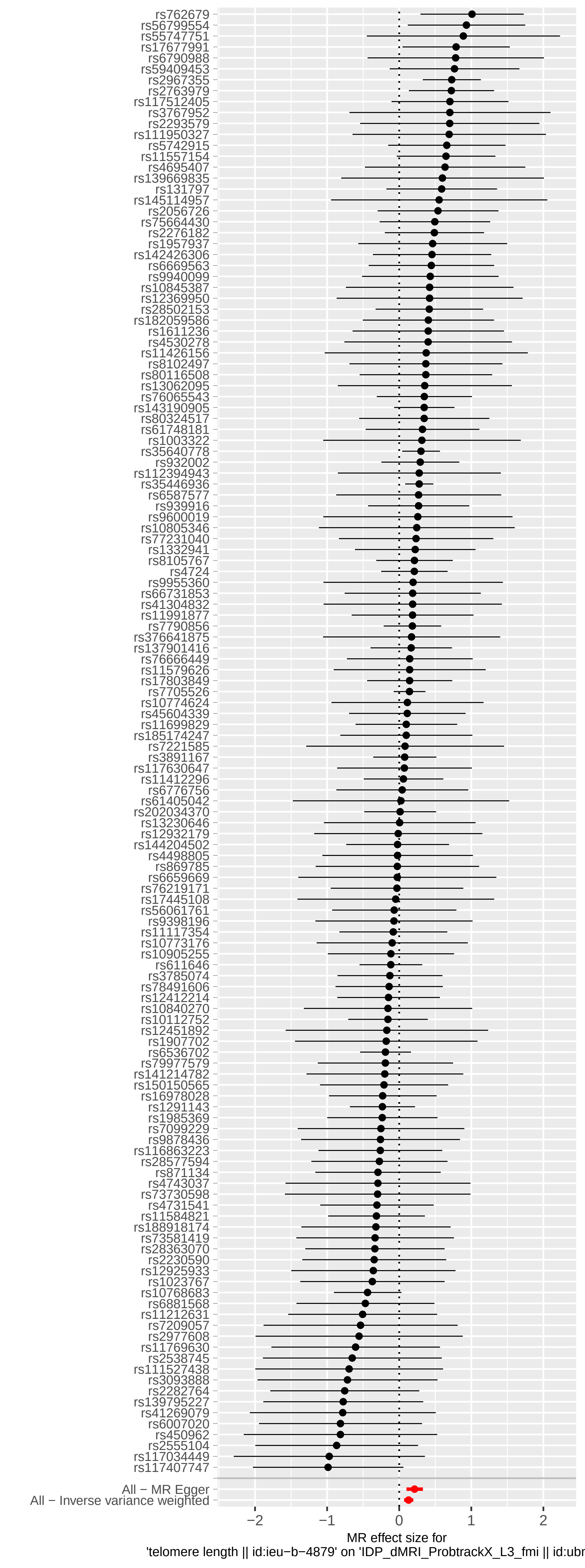
rs762679
rs56799554
rs55747751
rs17677991
rs6790988
rs59409453
rs2967355
rs2763979
rs117512405
rs3767952
rs2293579
rs111950327
rs5742915
rs11557154
rs4695407
rs139669835
rs131797
rs145114957
rs2056726
rs75664430
rs2276182
rs1957937
rs142426306
rs6669563
rs9940099
rs10845387
rs12369950
rs28502153
rs182059586
rs1611236
rs4530278
rs11426156
rs8102497
rs80116508
rs13062095
rs76065543
rs143190905
rs80324517
rs61748181
rs1003322
rs35640778
rs932002
rs112394943
rs35446936
rs6587577
rs939916
rs9600019
rs10805346
rs77231040
rs1332941
rs8105767
rs4724
rs9955360
rs66731853
rs41304832
rs11991877
rs7790856
rs376641875
rs137901416
rs76666449
rs11579626
rs17803849
rs7705526
rs10774624
rs45604339
rs11699829
rs185174247
rs7221585
rs3891167
rs117630647
rs11412296
rs6776756
rs61405042
rs202034370
rs13230646
rs12932179
rs144204502
rs4498805
rs869785
rs6659669
rs76219171
rs17445108
rs56061761
rs9398196
rs11117354
rs10773176
rs10905255
rs611646
rs3785074
rs78491606
rs12412214
rs10840270
rs10112752
rs12451892
rs1907702
rs6536702
rs79977579
rs141214782
rs150150565
rs16978028
rs1291143
rs1985369
rs7099229
rs9878436
rs116863223
rs28577594
rs871134
rs4743037
rs73730598
rs4731541
rs11584821
rs188918174
rs73581419
rs28363070
rs2230590
rs12925933
rs1023767
rs10768683
rs6881568
rs11212631
rs7209057
rs2977608
rs11769630
rs2538745
rs111527438
rs3093888
rs2282764
rs139795227
rs41269079
rs6007020
rs450962
rs2555104
rs117034449
rs117407747
All − MR Egger
All − Inverse variance weighted
−2
−1
0
1
2
MR effect size for
'telomere length || id:ieu-b-4879' on 'IDP_dMRI_ProbtrackX_L3_fmi || id:ubr
